# Iron Metabolism Genes, Low-Level Lead Exposure, and QT Interval

**DOI:** 10.1289/ehp.11559

**Published:** 2008-08-22

**Authors:** Sung Kyun Park, Howard Hu, Robert O. Wright, Joel Schwartz, Yawen Cheng, David Sparrow, Pantel S. Vokonas, Marc G. Weisskopf

**Affiliations:** 1 Department of Environmental Health Sciences, University of Michigan School of Public Health, Ann Arbor, Michigan, USA; 2 Department of Environmental Health, Harvard School of Public Health, Boston, Massachusetts, USA; 3 Channing Laboratory, Department of Medicine, Brigham and Women’s Hospital, Harvard Medical School, Boston, Massachusetts, USA; 4 College of Public Health, National Taiwan University, Taipei, Taiwan; 5 VA Normative Aging Study, Veterans Affairs Boston Healthcare System, and the Department of Medicine, Boston University School of Medicine, Boston, Massachusetts, USA

**Keywords:** gene–environment interaction, heme oxygenase-1, hemochromatosis, iron, lead, transferrin

## Abstract

**Background:**

Cumulative exposure to lead has been shown to be associated with depression of electrocardiographic conduction, such as QT interval (time from start of the Q wave to end of the T wave). Because iron can enhance the oxidative effects of lead, we examined whether polymorphisms in iron metabolism genes [hemochromatosis (*HFE*), transferrin (*TF*) C2, and heme oxygenase-1 (*HMOX-1*)] increase susceptibility to the effects of lead on QT interval in 613 community-dwelling older men.

**Methods:**

We used standard 12-lead electrocardiograms, K-shell X-ray fluorescence, and graphite furnace atomic absorption spectrometry to measure QT interval, bone lead, and blood lead levels, respectively.

**Results:**

A one-interquartile-range increase in tibia lead level (13 μg/g) was associated with a 11.35-msec [95% confidence interval (CI), 4.05–18.65 msec] and a 6.81-msec (95% CI, 1.67–11.95 msec) increase in the heart-rate–corrected QT interval among persons carrying long *HMOX-1* alleles and at least one copy of an *HFE* variant, respectively, but had no effect in persons with short and middle *HMOX-1* alleles and the wild-type *HFE* genotype. The lengthening of the heart-rate–corrected QT interval with higher tibia lead and blood lead became more pronounced as the total number (0 vs. 1 vs. ≥2) of gene variants increased (tibia, *p*-trend = 0.01; blood, *p*-trend = 0.04). This synergy seems to be driven by a joint effect between *HFE* variant and *HMOX-1* L alleles.

**Conclusion:**

We found evidence that gene variants related to iron metabolism increase the impacts of low-level lead exposure on the prolonged QT interval. This is the first such report, so these results should be interpreted cautiously and need to be independently verified.

Several epidemiologic studies have shown an association between lead exposure and cardiovascular disease. Recent reports using data from the Third National Health and Nutrition Examination Survey (NHANES III) suggest that even low levels of blood lead (< 10 μg/dL) are associated with an increased risk of death from all causes and from cardiovascular disease (Menke et al. 2006; Schober et al. 2006). A review of studies of lead exposure and cardiovascular disease concluded that there is sufficient evidence to infer a causal relationship of lead exposure with hypertension, and suggestive evidence of a causal relationship between lead and clinical cardiovascular outcomes and heart rate variability (Navas-Acien et al. 2007). Because iron can enhance the oxidative effects of lead, differences in iron metabolism could be a modifier of the adverse effects of lead on cardiovascular end points, as has been found for other end points (Wang et al. 2007; Wright et al. 2004).

Iron is a fundamental nutrient that plays a crucial role in vital biochemical activities, such as oxygen sensing and transport, electron transfer, and catalysis (Andrews 2000; Papanikolaou and Pantopoulos 2005). Under physiologic conditions, iron is bound to transferrin, the plasma iron carrier, which keeps iron soluble and nontoxic. Free iron can react with oxygen through the Fenton reaction to form dangerous free radicals that damage the macromolecular components of cells (Andrews 2000; Papanikolaou and Pantopoulos 2005). Oxidative stress caused by excessive free radicals may be associated with the pathogenesis of cardiovascular disease (Giordano 2005). Therefore, iron homeo stasis maintained by complex iron-transport and management systems is essential to prevent oxidative-stress–related health outcomes, and genetic polymorphisms affecting these systems could modify the effects of lead. Three specific iron metabolism genes that have variants known to alter protein function are hemochromatosis (*HFE*), transferrin (*TF*), and heme oxygenase-1 (*HMOX-1*). Two missense mutations of the *HFE* gene, C282Y and H63D, alter iron handling and are related to adult-onset hemochromatosis, an autosomal recessive genetic disease that increases absorption of ingested iron (Hanson et al. 2001). Transferrin is an iron-binding transport protein (Namekata et al. 1997b), and the *TF* C2 gene variant is a missense polymorphism associated with lower iron-binding capacity (Wong and Saha 1986). Heme oxygenase-1 is a heme-degrading enzyme that plays a critical role in regulating inflammatory and oxidative stress responses to iron (Balla et al. 2005; Maines 1988). A greater number of GT repeats in the *HMOX-1* gene promoter may reduce heme oxygenase-1 inducibility by reactive oxygen species (Yamada et al. 2000). Long alleles of the microsatellite polymorphism in *HMOX-1* have been associated with respiratory (Guenegou et al. 2006; Kikuchi et al. 2005; Yamada et al. 2000) and cardiovascular events (Chen et al. 2002; Funk et al. 2004).

We previously found a significant association between cumulative exposure to lead, as measured in bone, and prolonged QT interval (time from start of the Q wave to end of the T wave), a marker of sudden cardiac death risk and ventricular arrhythmia, in a community-based cohort of elderly men (Cheng et al. 1998). In the present study, we hypothesized that genetic polymorphisms related to iron metabolism modify the effect of low-level lead exposure on cardiac function. Therefore, we examined whether the associations between QT interval and lead levels in bone and blood are modified by *HFE*, *TF* C2, and *HMOX-1* genotypes, and whether there are joint effects among these iron metabolism genes.

## Materials and Methods

### Study population

Participants in our study were from the Normative Aging Study (NAS), a longitudinal study of aging initiated by the Veterans Administration in 1963, when 2,280 men from the Greater Boston, Massachusetts, area free of known chronic medical conditions were enrolled (Bell et al. 1966). Participants were asked to return for examinations, including electrocardiograms, every 3–5 years. Beginning in 1991, NAS participants who gave their informed consent were invited to undergo bone lead measurements. Blood lead was measured in participants’ blood specimens obtained during each subject’s visit. Of the 1,349 subjects who were seen for their regularly scheduled visits between August 1991 and December 1995, 783 participated in the bone lead study. No important differences were detected between participants and non-participants in the bone lead study (Cheng et al. 1998). We excluded 8 subjects with high bone lead measurement uncertainties (> 10 and > 15 μg/g for tibia and patella bones, respectively), 19 subjects missing QT-interval data, and 12 subjects with missing values of the potential confounding factors. Of the remaining 744 participants, 613 subjects were successfully genotyped for all three iron metabolism genes, *HFE*, *TF* C2, and *HMOX-1*.

### Bone lead and blood lead measurements

Bone lead levels were measured using a K-shell X-ray fluorescence (KXRF) instrument (ABIOMED, Danvers, MA, USA) at two sites: the midtibial shaft and the patella. The physical principles, technical specifications, and validation of this instrument have been described in detail elsewhere (Burger et al. 1990). The tibia and patella have been targeted for bone lead research because they consist mainly of pure cortical and pure trabecular bone, respectively, and thus represent the two main bone compartments. Lead in trabecular bone has a faster turnover rate and therefore reflects more recent exposure than that in the cortical bone. The KXRF instrument provides an unbiased estimate of bone lead levels (normalized for bone mineral contents as micrograms of lead per gram of bone mineral) and an estimate of the uncertainty associated with each measurement. Blood lead levels were analyzed using standard graphite furnace atomic absorption spectroscopy. The detection limit of this instrument was 1 μg/dL.

### QT-interval measurement

Standard resting 12-lead electrocardiograms taken at the same time as subjects’ bone lead and blood lead measurements were extracted and sent to the Minnesota ECG Coding Center. Tracings with any abnormalities were identified and classified according to the Minnesota coding system. We used the heart-rate–corrected QT intervals (QTc), calculated by Bazett’s formula, for analysis.

### HFE, TF *C2, and* HMOX-1 *genotyping.*

We extracted high-molecular-weight DNA from the white blood cells with commercially available PureGene Kits (Gentra Systems, Minneapolis MN, USA). Multiplex polymerase chain reaction (PCR) assays were designed using SpectroDESIGNER software (Sequenom, San Diego, CA, USA) by inputting sequence containing the single-nucleotide polymorphism (SNP) site and 100 bp of flanking sequence on either side of the SNP. For this assay, four SNPs were multiplexed: *HFE* RS1800562 and RS1799945, *TF* RS1049296, and *ALAD* RS1800435. For this study, we analyzed only the results for the *HFE* RS1800562 and RS1799945 and *TF* RS1049296 SNPs. We carried out multiplex PCR to generate short PCR products (> 100 bp) containing one SNP or insertion deletion. We used the following primers in the multiplex assay: *a*) *HFE* RS1800562: forward PCR primer (5′-ACGTTGGATGTACCCCAGATCA-CAATGAGG-3′), reverse PCR primer (5′-ACGTTGGATGTGGATAACCTT-GGCTGTACC-3′), extension primer (5′-GAAGAGCAGAGATATACGT-3′); *b*) *HFE* RS1799945: forward PCR primer (5′-ACGTTGGATGTCTACTG-GAAACCCATGGAG-3′), reverse PCR primer (5′-ACGTTGGATGTTGAAGC-TTTGGGCTACGTG-3′), extension primer (5′-GCTGTTCGTGTTCTATGAT-3′); *c*) *TF* RS1049296: forward PCR primer (5′-ACGTTGGATGTGAGTTGC-TGTGCCTTGATG-3′), reverse PCR primer (5′-ACGTTGGATGATCTTTCCGTG-TGACCACAG-3′), extension primer (5′-CGCATACTCCTCCACAG-3′).

**Figure 1:**
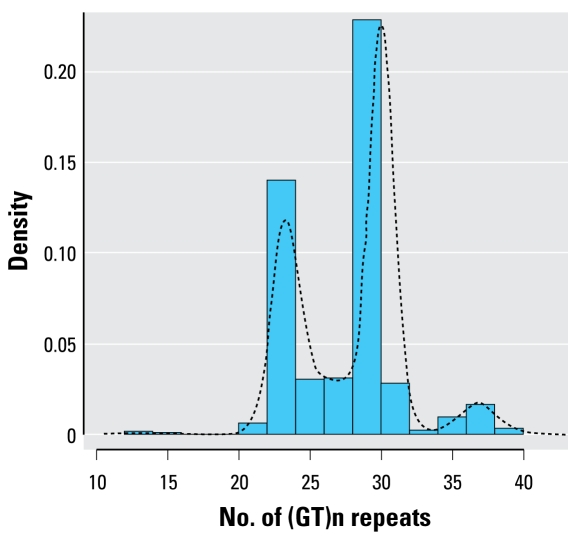
Distribution of the number of (GT)n repeats of the *HMOX-1* gene promoter (*n* = 613; 1,226 alleles).

The *HMOX-1* [UniGene accession number Hs.517581 (National Center for Biotechnology Information 2008)] micro satellite (GT)n-length assay was designed according to the method of Yamada et al. (2000). Briefly, the *HMOX-1* locus was amplified by PCR at the 5′ promoter flanking region containing (GT)n repeats using a fluorescently labeled sense primer (5′-AGAGCCTGCAGCTTCTCAGA-3′) and an unlabeled antisense primer (5′-ACAAAGTCTGGCCATAGGAC-3′). The sizes of the PCR products were analyzed with a laser-based automated DNA sequencer. The number of (GT)n repeats in the *HMOX-1* gene promoter ranged from 13 to 41. The distribution of (GT)n repeats was trimodal, with peaks at 23, 30, and 38 repeats (Figure 1). Therefore, we classified the alleles into three subclasses according to the number of (GT)n repeats, as previously reported (Guenegou et al. 2006; Hirai et al. 2003; Kikuchi et al. 2005): class S [short, < 27 (GT)n repeats], class M [medium, 27–32 (GT)n repeats], and class L [long, ≥33 (GT) n repeats]. The subjects were then classified as having L/L, L/M, L/S, M/M, M/S, or S/S genotypes. Although the exact cutoff for *HMOX-1* modulation is still unknown, many studies reported that L alleles are associated with the development of oxidative-stress–related diseases (Chen et al. 2002; Guenegou et al. 2006; Hirai et al. 2003; Kikuchi et al. 2005; Yamada et al. 2000).

### Statistical analysis

We performed allele and genotype frequencies and Hardy-Weinberg equilibrium tests for each genotype. Linear regression analyses were conducted to evaluate the association between QTc interval and lead biomarkers. We controlled for age (years), body mass index (BMI; kg/ m^2^), smoking status (never/former/current), and diabetes status (yes/no). We also controlled for albumin-adjusted serum calcium (milligrams per deciliter) because low serum calcium was associated with prolonged QTc interval among men in NHANES III (Benoit et al. 2005). Because lead exposure has been associated with hypertension (Hu et al. 1996; Navas-Acien et al. 2007) and possibly myocardial infarction (Menke et al. 2006; Schober et al. 2006), history of myocardial infarction and hypertension (yes/no) may be on the causal pathway between lead and QTc intervals. Therefore, our primary models did not contain these variables, but we compared these with models that included those variables. Finally, we compared models with and without a variable indicating beta-blocker use because they are used for long QT syndrome (Shimizu and Antzelevitch 1998), but this variable had many missing values.

To assess the potential modifying effects of *HFE*, *TF* C2, and *HMOX-1* genotype, we ran separate regression models stratified by those genotypes. We also ran regression models including a cross-product term for interaction between each genotype and each lead marker along with the main effects. To evaluate joint effects of these three genotypes, we combined the three genotypes into a single ordinal variable representing the number of gene variants (0 vs. 1 vs. 2 or 3). We also grouped the subjects into the combinations of three genotypes (2^3^: none, *HFE* only, *TF* C2 only, *HMOX-1* only, *HFE* and *TF* C2, *TF* C2 and *HMOX-1*, *HFE* and *HMOX-1*, and all three genotypes) and ran separate regression models stratified by these categories. To account for chance associations from multiple comparisons, we estimated the false-positive report probability (FPRP) for statistically significant gene–environment interaction findings using a modified method described by Wacholder et al. (2004), originally introduced in molecular/genetic epidemiology and now widely accepted in that literature. Although a *p*-value provides the probability of a given test statistic if the null hypothesis is true, the FPRP is the probability that an observed statistically significant association is a false positive. The FPRP value is determined by the *p*-value, the given prior probability for the association, and the statistical power of the test. We set an FPRP threshold of < 0.5 (i.e., ≤ 50% probability that the study hypotheses were falsely positive) as “noteworthy” for summary interactions; thus, FPRP values < 0.5 indicate significant interactions that remained robust for a given prior probability. We conducted statistical analyses using SAS (version 9.1; SAS Institute, Inc., Cary, NC, USA) and R (version 2.6.1; R Foundation for Statistical Computing 2003) software systems. All probability values are two tailed (α = 0.05).

## Results

Table 1 shows the frequencies, bone and blood lead levels, and QTc interval by *HFE*, *TF* C2, and *HMOX-1* genotypes. The *HFE* C282Y genotype frequencies were wild type (CC), 85.5%; heterozygote (CY), 13.9%; and homozygote (YY), 0.7%. Frequencies of *HFE* H63D genotypes were wild type (HH), 77.2%; heterozygote (HD), 20.2%; and homo zygote (DD), 2.6%. The frequencies of *TF* C2 were C2 negative, 68.7%; heterozygote, 28.2%; and homozygote, 3.1%. Seventy-four subjects (12.1%) were L-allele (L/L, L/M, L/S) carriers. When collapsed into a single variable indicating the presence or absence of *HFE* and *TF* C2 mutations, 216 (35.2%) and 192 (31.3%) subjects carried at least one copy of *HFE* variant and *TF* C2 variants, respectively. The distributions of all genotypes were in Hardy-Weinberg equilibrium (C282Y: χ^2^ = 0.07, *p* = 0.79; *TF* C2: χ^2^ = 0.46, *p* = 0.50; *HMOX-1*: χ^2^ = 5.59, *p* = 0.13), except H63D (χ^2^ = 4.88, *p* = 0.03), but the overall prevalence for H63D genotypes among those who were genotyped in the NAS cohort (*n* = 1073) was in Hardy-Weinberg equilibrium (χ^2^ = 2.34, *p* = 0.13). Subjects who were homozygous for C282Y had higher bone lead levels, and those who were homozygous for H63D and heterozygous for both C282Y and H63D (compound heterozygotes) had lower bone lead levels. There were no differences in bone or blood lead levels by *TF* C2 or *HMOX-1* genotype. QTc intervals did not differ by each genotype.

Table 2 presents distributions of characteris tics for the NAS men in our study sample (all three genes successfully genotyped; *n* = 613) and those men excluded (missing genotype data; *n* = 131). The men in our study sample were slightly younger, had lower tibia and patella bone lead levels, and were less likely to have a history of myocardial infarction or to have ever smoked than those excluded. Among those in our study sample, subjects with two or three gene variants had slightly lower BMI and patella lead levels, and slightly more had never smoked, compared with those with zero or one gene variant, but otherwise were similar.

We examined the effects of iron metabolism genes on QTc interval independent of lead exposures (data not shown). After controlling for potential confounders, no iron gene variant alone was significantly associated with QTc interval. Compared with subjects with no gene variants, those with one of any of the three gene variants had a borderline significantly lower QTc interval [−4.93 msec; 95% confidence interval (CI), −10.10 to 0.23 msec], but we found no difference in QTc intervals among those with two or three gene variants.

In a model including all 613 subjects, higher tibia lead and patella lead levels were significantly associated with increased QTc interval after adjustment for potential confounders (Table 3). Blood lead levels were also positively associated with QTc interval, but not significantly so. In stratified analyses by *HFE*, *TF* C2, or *HMOX-1* genotypes, the associations of all lead biomarkers with QTc interval were stronger in persons with gene variants, although the interaction by genotype was statistically significant only for tibia lead and the *HMOX-1* variants. A one-interquartile-range (IQR) increase in tibia lead level (13 μg/g) was associated with a 1.60-msec (95% CI, −1.12 to 4.32 msec) longer QTc interval among subjects without L *HMOX-1* alleles and an 11.35-msec (95% CI, 4.05 to 18.65 msec) longer QTc interval among subjects with any L allele, which was statistically significantly different (*p* for interaction = 0.01; Table 3). Among subjects with either *HFE* variant, a one-IQR increase in tibia lead level was associated with a 6.81-msec (95% CI, 1.67 to 11.95 msec) longer QTc interval, but although this was a significant and much larger effect size than for subjects with wild-type *HFE*, the difference from those with wild-type *HFE* was not statistically significant. When we compared individuals with C282Y variant (C282Y homozygotes and heterozygotes, *n* = 89) with those without the C282Y variant (i.e., ignoring H63D status; *n* = 524), the difference was borderline significant (*p* for interaction = 0.06; data not shown).

We also found increasing trends in the lengthening of the QTc interval with higher levels of tibia lead (*p*-trend = 0.01) and blood lead (*p*-trend = 0.04) as the overall number of any of the gene variants increased. Results for patella lead were similar but did not reach statistical significance.

We calculated FPRP to evaluate whether the observed significant interactions between tibia lead and both *HMOX-1* and the number of the gene variants might be false positives (data not shown). No FPRP values were less than 0.5 even with a high prior probability (e.g., prior *p* = 0.25), indicating that none of the significant gene–lead interaction results reached the 0.5 FPRP level criterion for being noteworthy.

**Figure 2:**
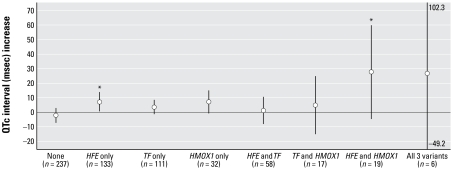
Adjusted parameter estimates and 95% CIs of QTc interval in relation to an IQR increase in tibia lead levels (13 μg/g) stratified by the combinations of *HFE*, *TF* C2, and *HMOX-1* genotypes. All models were adjusted for age, BMI, and smoking status, but non significant covariates (albumin-adjusted serum calcium and diabetes status) used in Table 3 were omitted because the number of subjects with all three variants was too small (*n* = 6) to estimate β-coefficients. The results with and without these covariates were very similar in other categories. The numbers next to the upper and lower tails of “All three variants” group indicate that the 95% CIs exceed the range of the *y*-axis. *p for interaction < 0.05 compared with participants with no variants (None group).

Joint effects between iron metabolism genotypes on the association between tibia lead levels and QTc interval are shown in Figure 2. The association among carriers of *HFE* alone was significantly different from that among participants with no variants (*p* for interaction = 0.03). Carriers of *HMOX-1* L alleles alone had a borderline significant lengthening of QTc intervals with increasing tibia lead (*p* for interaction = 0.08). Among carriers of both *HFE* variant and *HMOX-1* L alleles, the lead-related lengthening of the QTc interval (27.64 msec; 95% CI, −4.21 to 59.49 msec; *n* = 19) was substantially longer than the sum of the individual associations with either of those variants alone, and the effect of tibia lead in this group was significantly different compared with those with no variants (*p* for interaction = 0.01).

The overall trends with patella lead and blood lead were similar to but less significant than those with tibia lead (data not shown). Further adjustment for hypertension, myocardial infarction, and/or beta-blocker medication did not change the results (data not shown).

## Discussion

We found that *HFE* and *HMOX-1* genotypes modified the relationship between low-level lead exposure, particularly lead in tibia bone, and an increase in QTc interval in older men. Intriguingly, subjects with both *HFE* variants and *HMOX-1* L alleles had a particularly large effect estimate for the association between tibia lead and QTc interval, suggesting a possible synergy between *HFE* variant and *HMOX-1* L alleles in modifying the association between lead exposure and cardiac toxicity. There was less evidence for effect modification by *TF* C2 genotype. Results for blood lead were similar to but less significant than those for tibia lead.

This is the first study to evaluate effect modification by iron metabolism genes either individually or jointly on the association between low-level lead exposure and prolonged QTc interval. Although our study is not a comprehensive assessment of iron metabolism genes and lead toxicity, it is a logical first step in determining whether iron metabolism gene variants modify lead toxicity. Each of the gene variants we meas ured increase intra cellular iron (*HFE* variants), are associated with a disease of increased oxidation (*TF* C2), or are associated with increased oxidative cellular toxicity (*HMOX-1* L alleles). The commonality among these genes of increasing iron-induced oxidative toxicity make them of interest not only individually but also in combination. Interactions between iron metabolism and lead toxicity may result from enhanced oxidative processes when excess iron and lead co-occur.

The functional defects of the C282Y and H63D HFE variant proteins result in iron overload (Hanson et al. 2001). In the presence of excess iron, exogenous oxidative toxins, such as lead, may trigger the Fenton reaction, enhancing oxidative stress. In fact, lipid peroxidation by lead is increased in the presence of iron (Adonaylo and Oteiza 1999). Therefore, persons with these *HFE* variants might be expected to suffer from an enhanced effect of lead on oxidative-stress–related health outcomes, including QTc interval. Lead toxicity affects multiple tissues, and similar oxidative toxicity may occur in tissues other than the heart. A recent study conducted in the same NAS cohort showed that individuals with at least one copy of *HFE* variant had steeper cognitive decline compared with wild-type carriers (Wang et al. 2007), which supports this hypothesis.

Heme oxygenase-1 is a stress-response protein that is induced by a variety of oxidative stimuli (Maines 1988). Heme oxygenase-1 lowers levels of free iron in cells, which is an important mechanism of its protection against oxidative stress. Long alleles of the microsatellite polymorphism in *HMOX-1* are associated with lower protein expression levels (Yamada et al. 2000), and therefore higher oxidative stress and related sequelae (Chen et al. 2002; Funk et al. 2004; Guenegou et al. 2006; Kikuchi et al. 2005; Yamada et al. 2000). Lead has been found to induce heme oxygenase-1 both *in vitro* and *in vivo* (Cabell et al. 2004; Korashy and El-Kadi 2004; Vargas et al. 2003). Reduced induction of heme oxygenase-1 by lead among subjects with *HMOX-1* L alleles would result in increased oxidative stress and could explain the greater prolongation of the QTc interval from lead exposure in this group.

The *TF* C2 gene product has been associated with lower total iron-binding capacity (Wong and Saha 1986), which has been proposed to lead to excess free iron (Robson et al. 2004). As in the case of *HFE*, in the presence of excess iron, lead may trigger the Fenton reaction and enhance oxidative stress. This mechanism has been proposed to explain the association between *TF* C2 and Alzheimer’s disease, which involves increased oxidative toxicity in the brain (Loeffler et al. 1995; Namekata et al. 1997a). It is unclear why carrying a *TF* C2 variant with either *HMOX-1* L alleles or one of the *HFE* variants was associated with a shorter lead-related QTc interval increase compared with those with wild-type *TF* and one of the other variants. This could be the result of chance, but it raises the possibility that the *TF* C2 variant could be protective for the effects of *HMOX-1* L alleles or the *HFE* variants on the lead–QTc association.

In our previous study (Cheng et al. 1998), the associations between bone lead levels and QTc interval were observed only in men < 65 years, not in men ≥ 65 years of age. In that study, it was proposed that this might be due to an increased number of causes of cardiac conduction disturbances in the older group that may mask the impact of lead exposure. Given those results, an alternative possibility for the present findings could be that the observed gene–lead interactions result from an unbalanced distribution of genotype by age. However, the proportions of those < 65 years by genotype [*HFE* (wild-types, 37.7%, vs. variants, 38.6%), *TF* C2 (wild-types, 36.3%, vs. variants, 41.5%), and *HMOX-1* (any L allele, 41.0%, vs. others, 37.0%)] were very similar, and thus the gene–lead interactions we report here are likely not simply a proxy for age interactions. We could not evaluate two-way interactions by iron genes and age because of the small sample size in each category.

The present study has several limitations. We cannot rule out that the observed significant interactions may be chance findings related to the moderate sample size. Kraft and Hunter (2005) indicated that most “significant” interactions of gene and environment will be false positives with sample sizes in the order of a few hundred, and our FPRP analysis raises concern that this could be the case with the present findings. Although our study is cross-sectional, because bone lead is a marker of long-term cumulative exposures to lead, the observed associations would be temporally relevant. Our study subjects were predominantly white men, 95% of whom were of European descent, so the present findings may not be generalizable to women or to other racial/ethnic groups. On the other hand, the homogeneity of our population improves internal validity of our results and makes it unlikely that population stratification could explain our findings. As with all observational studies, there is the possibility that residual and unmeasured confounding factors may have affected our results. We were, however, able to account for many known risk factors for prolonged QTc interval, such as age, BMI, cigarette smoking, serum calcium, and history of myocardial infarction, hypertension, and diabetes. Therefore, the observed associations are unlikely to be explained by these factors. Serum iron parameters, such as ferritin and transferrin, were not available in this cohort.

In conclusion, our results suggest that *HMOX-1* L-alleles and C282Y and H63D *HFE* variants—genes involved in iron metabolism—may confer susceptibility to cardiac toxicity from lead exposure. They also raise the possibility that polymorphisms in iron metabolism genes could also modify susceptibility to other factors that can interact with iron to produce oxidative stress, such as exposure to other transition metals. This is the first such report, so these results should be interpreted cautiously and need to be independently verified.

## Figures and Tables

**Table 1:** Genotype frequencies, bone and blood lead levels, and QTc interval [median (IQR)] by *HFE*, *HMOX-1*, and *TF* C2 genotype (*n* = 613).

Genotype	No. (%)	Tibia lead (μg/g)	Patella lead (μg/g)	Blood lead (μg/dL)	QTc interval (msec)
*HFE*					
Wild-type (CC/HH)	397 (64.8)	20 (14–28)	27 (18–39)	5 (4–7.5)	394 (376–414)
C282Y heterozygotes (CY/HH)	72 (11.8)	19.5 (14–26.5)	24.5 (17.5–35.5)	5 (3.7–8)	396 (377–410)
C282Y homozygotes (YY/HH)	4 (0.7)	35 (29.5–37.5)	42 (28–62)	7.5 (5.5–8)	410 (405–429)
Compound heterozygotes (CY/HD)	13 (2.1)	15 (9–15)	22 (19–33)	4 (3–5.5)	378 (373–395)
H63D heterozygotes (CC/HD)	111 (18.1)	19 (14–27)	25 (18–37)	5 (3–7)	393 (374–413)
H63D homozygotes (CC/DD)	16 (2.6)	17 (12.5–21.5)	24 (18.5–30.5)	5 (4–7)	389 (375–405)
Presence of either *HFE* variant	216 (35.2)	19 (14–26)	25 (18–36)	5 (3–7)	393 (376–411)
*TF* C2					
Wild-type (−/−)	421 (68.7)	19 (14–27)	27 (18–38)	5 (3.7–7)	394 (377–413)
C2 heterozygotes (−/+)	173 (28.2)	19 (13–28)	25 (18–36)	5 (3.7–7)	395 (376–410)
C2 homozygotes (+/+)	19 (3.1)	20 (14–26)	23 (15–40)	5 (4–9)	383 (370–412)
Presence of *TF* C2 variant	192 (31.3)	19 (13–27)	24 (17–36)	5 (3.8–8)	394 (374–411)
*HMOX-1*^a^					
S/S	85 (13.9)	20 (13–28)	27 (18–42)	5 (4–7)	394 (376–412)
M/S	242 (39.5)	19 (14–26)	27 (18–37)	5 (3.7–8)	393 (376–413)
M/M	212 (34.6)	19.5 (13–27)	24.5 (17–37.5)	5 (3.4–7)	396 (376–413)
L/S	28 (4.6)	18.5 (13.5–31)	24.5 (19–36)	5 (4–7.5)	387 (371–407)
L/M	44 (7.2)	19.5 (12.5–27)	24.5 (21–38.5)	5 (3.5–7)	402 (377–413)
L/L	2 (0.3)	19 (19–19)	33.5 (32–35)	7 (6–8)	362 (334–390)
Any L allele	74 (12.1)	19 (13–30)	25 (20–36)	5 (4–7)	390 (376–410)

a S, short alleles (< 27 GT repeats) of *HMOX-1*; M, medium alleles (27–32 GT repeats) of *HMOX-1*; L, long alleles (≥ 33 GT repeats) of *HMOX-1*.

**Table 2:** Characteristics of study population by the number of gene variants.

		No. of gene variants			
Characteristic	Subjects with all three genotypes	0	1	2 or 3	Subjects excluded^a^
No.	613	237	276	100	131
Continuous variables (mean ± SD)					
Age (years)	67.3 ± 7.2	67.6 ± 7.3	67.3 ± 7.2	66.6 ± 7.0	70.0 ± 7.3
BMI (kg/m^2^ )	27.9 ± 3.7	28.0 ± 3.8	28.1 ± 3.6	27.2 ± 3.5	27.6 ± 4.5
Albumin-adjusted serum calcium (mg/dL)	9.0 ± 0.3	9.0 ± 0.3	9.0 ± 0.3	9.0 ± 0.4	9.1 ± 0.4
Systolic blood pressure (mm Hg)	137.1 ± 18.1	137.0 ± 18.5	136.9 ± 17.1	137.7 ± 20.0	137.0 ± 17.1
Diastolic blood pressure (mm Hg)	81.7 ± 9.7	81.7 ± 10.0	81.5 ± 9.5	82.4 ± 9.5	80.6 ± 9.5
QTc interval (msec)	395.7 ± 30.3	398.1 ± 31.7	393.3 ± 28.2	396.5 ± 32.3	400.2 ± 38.7
Categorical variables [*n* (%)]					
Smoking status					
Never	195 (31.8)	67 (28.3)	92 (33.3)	36 (36.0)	29 (22.1)
Former	371 (60.5)	151 (63.7)	163 (59.1)	57 (57.0)	91 (69.5)
Current	47 (7.7)	19 (8.0)	21 (7.6)	7 (7.0)	11 (8.4)
History of myocardial infarction	29 (4.7)	10 (4.2)	14 (5.1)	5 (5.0)	12 (9.2)
Diabetes	43 (7.0)	14 (5.9)	22 (8.0)	7 (7.0)	14 (10.7)
Hypertension	266 (43.4)	108 (45.6)	111 (40.2)	47 (47.0)	53 (40.5)
Lead levels [median (IQR)]					
Tibia lead (μg/g)	19 (14–27)	19 (13–27)	20 (14–28)	19 (12–26)	23 (16–29)
Patella lead (μg/g)	26 (18–37)	27 (17–39)	26 (18–37)	24 (19–35)	31 (21–43)
Blood lead (μg/dL)^b^	5 (4–7)	5 (4–7)	5 (4–7)	5 (3–7)	5 (4–7.5)

QTc intervals = QT measured in lead I × √ (heart rate/60).

a Subjects excluded because of missing genotype data.

b Reduced numbers of subjects with blood lead for subjects with all three genotypes, 0 gene variants, 1 gene variant, and 2 or 3 gene variants were 599, 232, 267, 100, and 128, respectively.

**Table 3:** Adjusted parameter estimates^a^ for the difference in QTc interval per IQR increase in lead bio-marker level, stratified by *HFE*, *TF* C2, and *HMOX-1* genotypes.

	Tibia lead (IQR = 13 μg/g)		Patella lead (IQR = 19 μg/g)		Blood lead (IQR = 3 μg/dL)	
Parameter	β	95% CI	β	95% CI	β	95% CI
All subjects (*n* = 613)	2.85	0.29 to 5.40	2.64	0.13 to 5.15	1.30	−0.76 to 3.36
Stratified by iron metabolism genes						
*HFE*						
Wild type (*n* = 419)	1.83	−1.17 to 4.83	2.35	−0.63 to 5.33	0.50	−1.89 to 2.89
*HFE* variants (*n* = 228)	6.81	1.67 to 11.95	4.00	−0.97 to 8.97	4.42	−0.06 to 8.91
*p* for interaction^b^		0.11		0.58		0.14
* TF* C2						
C2 negative (*n* = 443)	1.97	−1.44 to 5.39	1.61	−1.74 to 4.97	0.12	−2.41 to 2.64
C2 homo/heterozygotes (*n* = 205)	3.56	−0.32 to 7.44	4.08	0.30 to 7.86	3.27	−0.31 to 6.86
*p* for interaction^b^		0.55		0.35		0.17
*HMOX-1*						
S or M alleles (*n* = 579)	1.60	−1.12 to 4.32	1.88	−0.83 to 4.59	1.20	−0.96 to 3.36
Any L allele (*n* = 83)	11.35	4.05 to 18.65	7.21	0.35 to 14.06	5.09	−1.89 to 12.08
*p* for interaction^b^		0.01		0.14		0.27
No. of gene variants						
0 (*n* = 237)	−2.34	−7.05 to 2.37	−0.53	−5.23 to 4.18	−0.82	−4.35 to 2.71
1 (*n* = 276)	5.17	2.02 to 8.32	3.77	0.57 to 6.98	2.65	−0.66 to 5.96
2 or 3 (*n* = 100)	7.26	−0.72 to 15.25	5.38	−1.74 to 12.49	6.71	−0.70 to 14.13
*p* for trend^b^	0.01		0.10		0.04	

a Adjusted for age, BMI, albumin-adjusted serum calcium, smoking status, and diabetes status.

b *p*-Values for interaction and trend between lead biomarker and genotype were computed from the models including cross-product terms between genotype and all other covariates analogous to the stratified models.
